# Case Report: Delayed-type hypersensitivity reaction to meropenem in an elderly patient—successful management with imipenem-cilastatin

**DOI:** 10.3389/fmed.2025.1671455

**Published:** 2025-10-29

**Authors:** Cuilin He, Maozhu Liu, Jiao Ye, Hong Zheng, Junjie Yang, Lingmei Huang

**Affiliations:** ^1^Department of Pharmacy, The First People's Hospital of Shuangliu District (West China Airport Hospital of Sichuan University), Chengdu, China; ^2^Center of Infectious Diseases, West China Hospital, Sichuan University, Chengdu, China; ^3^Department of Clinical Laboratory, People's Hospital of Deyang City, Deyang, China; ^4^Department of Pharmacy, West China Hospital, Sichuan University, Chengdu, China

**Keywords:** meropenem, adverse drug reaction, delayed-type hypersensitivity reaction, imipenem-cilastatin, cross-reactivity

## Abstract

Meropenem is a clinically essential carbapenem antibiotic with a broad antimicrobial spectrum, potent antibacterial efficacy, and high stability against *β*-lactamase, which plays a pivotal role in anti-infective therapy. In clinical practice, meropenem generally shows a favorable safety and tolerability profile, with relatively few reports of serious adverse reactions. Here, we present a case of an 80-year-old woman who developed a delayed-type hypersensitivity (DTH) reaction after receiving anti-infective treatment with meropenem. This allergic reaction was immediately managed with calcium gluconate, dexamethasone sodium phosphate, and ebastine. Meanwhile, meropenem was discontinued and replaced with imipenem-cilastatin to control the infection. Following a 14-day antimicrobial therapy, initially with imipenem-cilastatin and subsequently with amoxicillin-clavulanate potassium tablets, the patient’s skin rashes gradually subsided and the infection was successfully controlled. This case highlights the risk of DTH reaction induced by meropenem in clinical settings. Clinicians should remain vigilant for DTH reactions during meropenem therapy and cautiously consider alternative carbapenems such as imipenem-cilastatin.

## 1 Introduction

Meropenem is a dehydropeptidase-1 stable carbapenem antibiotic with broad-spectrum antibacterial activity against various gram-positive and gram-negative organisms. Mechanistically, meropenem exerts its bactericidal effect by penetrating the bacterial outer membrane and irreversibly binding to penicillin-binding proteins (PBPs), thereby inhibiting peptidoglycan synthesis, and ultimately leading to cell death and lysis. Approved by the U.S. Food and Drug Administration (FDA) in 1996, meropenem is now widely used for treating multiple infectious diseases, such as septicemia, nosocomial pneumonia, complicated intra-abdominal infections, and urinary tract infections (1). Notably, a key advantage of meropenem over other carbapenems is its reduced tendency to induce seizures, making it a preferred choice for managing bacterial meningitis. Furthermore, clinical practice has confirmed the high safety profile of meropenem, with common adverse reactions including diarrhea, vomiting, abnormal liver enzymes, and skin rashes (2–4). Due to its favorable safety and potent antibacterial activity, meropenem remains an essential therapeutic option in modern antimicrobial therapy.

Delayed-type hypersensitivity (DTH) reactions are T-cell-mediated immune responses that typically develop days to weeks after antigen exposure (5). The current literature suggests low incidence of carbapenem-induced hypersensitivity ranging from 0.3–3.7% (6, 7), and carbapenem-induced DTH were only reported in rare case reports (8–12). However, when these reactions occur, they may progress to life-threatening severity, making rapid recognition and immediate intervention critical. Here, we report a DTH associated with meropenem, which was clinically manifested by erythematous morbilliform rashes 60 h post-administration. After receiving anti-allergic treatment and switching to imipenem-cilastatin for anti-infective therapy, the patient recovered completely and was subsequently discharged in stable condition. This case highlights the need for continued monitoring for delayed hypersensitivity reaction throughout meropenem therapy.

## 2 Case presentation

The patient’s disease progression and management timeline is shown as the Supplementary Figure S1. On February 27, 2025, an 80-year-old female patient with no known drug allergies was admitted for head injuries sustained in a fall 3 h earlier. The patient had a history of occasional elevated blood pressure, but managed without pharmaceutical intervention. She was subsequently diagnosed with a severe closed craniocerebral injury and received treatment at our hospital. Upon arrival, physical examination revealed elevated blood pressure (152/74 mmHg), decreased oxygen saturation (90%) with heart rate (65 beats/min), respiratory rate (14 breaths/min), and temperature (36.5 °C) within normal limits.

**Figure 1:**
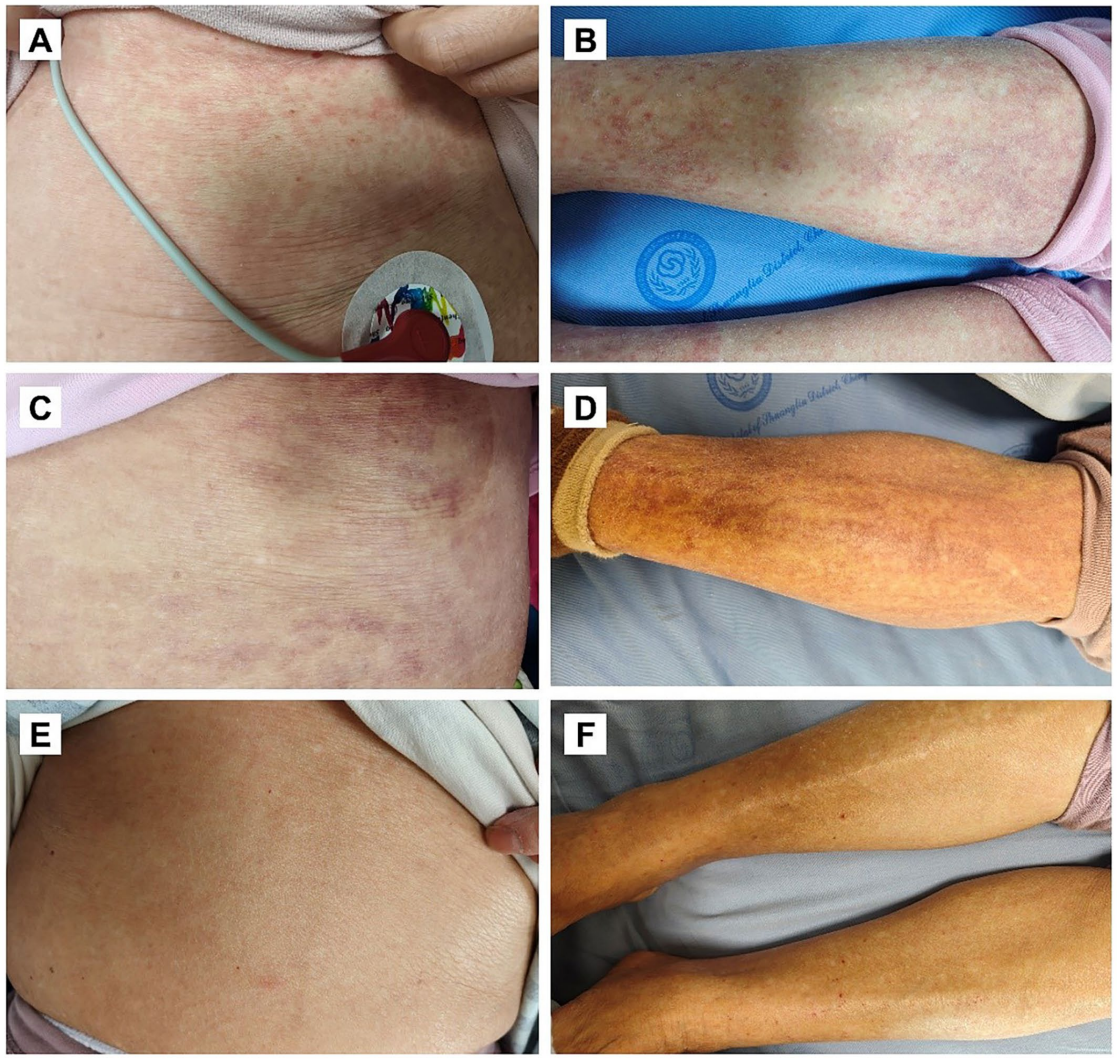
Clinical progression and treatment response of DTH. **(A,B)** The patient presented with generalized erythematous morbilliform rashes on March 26 (Day 28), with the most severe lesions on the abdomen **(A)** and bilateral lower limbs **(B)**. **(C,D)** After appropriate intervention, clinical improvement was observed on March 27 (Day 29), with partial resolution of skin rashes on the abdomen **(C)** and bilateral lower limbs **(D)**. **(E,F)** The skin rash was completely controlled by April 8 (Day 41), indicating satisfactory therapeutic response.

On February 28, a chest CT scan showed pulmonary infections and piperacillin tazobactam (4.5 g, intravenous drip, every 8 h) after negative skin test was used to control infection. Subsequently, the patient experienced a progression of the infection on March 19, manifesting as fever with chills (maximum temperature 39 °C), elevated procalcitonin (PCT) (6.85 ng/mL), and decreased white blood cell count (WBC 1.0 × 10^9^/L) and elevated C-reactive protein (CRP 62.62 mg/L). However, the chest CT scan suggested improved pulmonary lesions, with head imaging and urinalysis showed no abnormalities. These findings, when combined with the clinical presentation, definitively excluded pulmonary, intracranial, or urinary tract infections as potential contributors to disease progression. Consequently, blood infection was considered the most probable diagnosis, and antimicrobial therapy was changed to meropenem (1.0 g, intravenous drip, every 8 h) to optimize infection control. The patient’s temperature returned to normal after 24 h treatment, and infection-related indicators (WBC 3.68 × 10^9^/L) also recovered significantly (Table 1). 60 h after the first infusion of meropenem (March 26), the patient developed generalized erythematous morbilliform rashes accompanied by pruritus, initially involving the abdomen and then the back, arms, and legs (most prominent on the abdomen and both lower limbs) (Figure 1). After evaluating the patient’s drug exposures, a delayed hypersensitivity reaction secondary to meropenem treatment was identified as the most probable etiology. Calcium gluconate (1.0 g, intravenous injection, once a day), dexamethasone sodium phosphate (10 mg, intravenous drip, once a day), and ebastine (20 mg, oral, once a day) were immediately used to manage the allergic reaction, and meropenem was discontinued. On March 27, the patient’s skin rashes showed mild improvement. However, the patient again developed fever (maximum temperature 38.9 °C) with PCT above the upper range (1.19 ng/mL) and WBC below the lower range (2.91 × 10^9^/L). Given high suspicion of bloodstream infection, meropenem’s efficacy, and the patient’s prolonged antibiotic use predisposing to resistant bugs such as ESBLs-producing Gram-negative bacteria, imipenem-cilastatin (0.5 g, intravenous drip, every 8 h) was administered cautiously. After 3 days of treatment, the patient’s temperature returned to normal, and indicators such as PCT (0.47 ng/mL) and CRP (22.88 mg/L) improved. Following meropenem discontinuation and anti-allergic therapy, her skin rashes and pruritus gradually improved and completely disappeared on April 8 (Figure 1). Consequently, ebastine was discontinued (calcium gluconate and dexamethasone sodium phosphate were discontinued on March 29 and April 2, respectively). With satisfactory infection control achieved (maximum temperature 36.9 °C, WBC 3.46 × 10^9^/L, CRP 49.98 mg/L), intravenous imipenem-cilastatin was replaced by oral amoxicillin-clavulanate potassium tablets on April 9 (1.0 g orally, every 12 h). After completing a 14-day antimicrobial regimen consisting of imipenem-cilastatin followed by amoxicillin-clavulanate potassium tablets, the patient was discharged from the hospital and continued to take oral amoxicillin-clavulanate potassium tablets to prevent infection recurrence. At the two-month follow-up after discharge, clinical evaluation demonstrated that the patient’s infection and skin rashes were satisfactorily controlled with no other complications.

**Table 1:** Results of laboratory testing after admission.

Indicator	Reference ranges	2.27	3.1	3.2	3.9	3.10	3.16	3.19	3.23	3.4	3.27	3.30	4.6	4.11
WBC	3.5–9.5 × 10^9^/L	15.39	10.19	11.13	14.09	14.84	5.96	3.61	1.0	3.68	2.91	12.91	3.46	3.93
NEUT%	40–75%	91.1	88.2	88.9	79	64.9	74.3	66.6	55.1	39.2	48	80.8	53.3	47.8
PCT	0–0.046 ng/mL	–	–	–	–	1.65	–	–	6.85	-	1.19	0.47	-	-
CRP	0–10 mg/L	16.51	–	–	35.09	–	–	–	62.62	-	29.61	22.88	49.98	33.97

WBC, White blood cell; NEUT%, Percentage of neutrophilic granulocyte; PCT, Procalcitonin; CRP, C-reactive protein.

## 3 Discussion

DTH is an immune response orchestrated by antigen-specific effector T cells, representing a frequently observed adverse drug reaction in clinical practice (5). Unlike antibody-mediated immediate hypersensitivity reactions, DTH relies on T-cell antigen recognition, cytokine release, and subsequent tissue damage (13). This complex immunological cascade develops progressively and usually requires days to weeks to manifest, highlighting the importance of careful monitoring following drug exposure (5). Typically, DTH can occur in multiple organ systems such as skin, lungs, liver, and kidney. The skin is the organ most frequently affected by DTH, likely attributable to its abundant resident T cell population. The clinical manifestations of skin DTH are diverse, ranging from self-limiting conditions such as fixed drug eruption (FDE) and maculopapular exanthema (MPE) to life-threatening toxic epidermal necrolysis (TEN) and acute generalized exanthematous pustulosis (AGEP) (14). Drugs are significant causative factors of DTH, especially skin DTH reactions (5). While antibiotic-induced DTH was well-documented in the literature, meropenem-associated DTH remains an uncommon clinical occurrence. We report a meropenem-induced DTH case, underscoring the importance of DTH monitoring during meropenem therapy. Furthermore, the case offers references for appropriate treatment for allergic reactions and infections in such situation.

The patient in this case had no prior history of drug allergies and experienced no adverse drug reactions during the initial 23 days of hospitalization. However, 60 h after receiving meropenem therapy for infection treatment, the patient developed significant skin allergic manifestations. Considering that meropenem was the only newly introduced drug and no other therapeutic changes were made, meropenem was strongly suspected as the causative agent. The 60-h latency period between drug administration and symptom onset was consistent with the temporal characteristics of DTH. Thus, the patient was ultimately diagnosed with meropenem-induced skin DTH. In accordance with the European Academy of Allergy and Clinical Immunology (EAACI) guidelines (15), meropenem was promptly discontinued and anti-allergic treatment was administered. As a result, the patient’s allergic symptoms were alleviated.

To date, clinical research on meropenem-induced DTH is scarce. As summarized in Table 2, Noguerado-Mellado et al. (9) reported a case of a meropenem-induced DTH in a 61-year-old female patient who developed generalized scaly erythematous rashes involving oral, vaginal, and rectal mucosa progressing to desquamation 3–4 days after meropenem administration. Similarly, Morgado et al. (10) described a 38-year-old female patient who developed morbilliform eruption 10 days after meropenem administration, and another 61-year-old female patient exhibited maculopapular exanthema 11 days after receiving meropenem. Combined with our findings, these cases suggest that meropenem-associated DTH is a non-negligible clinical concern, revealing the imperative for both clinical monitoring and further mechanistic investigations to elucidate the underlying pathogenesis. Notably, all previously reported cases, including the present one, involved female patients, presented with skin or mucosal rashes, which indicates a certain degree of consistency of meropenem-induced DTH. However, our case presents several distinctive characteristics. Firstly, the rash in our patient appeared shortly after meropenem initiation, which contrasts with the typical latency period of one to several weeks for such reactions (5). This finding, however, aligns with prior observations by Blanca et al. (9), who reported cases of meropenem-induced generalized scaly erythematous rash that appeared within 7 days, suggesting that early onset can occur. Secondly, the extent of the rash in our case was more widespread. Additionally, in our case, eosinophil elevated (0.65 × 10^9^/L) when rash appeared on March 26 and returned to normal (0.17 × 10^9^/L) after anti-allergy treatment on April 6, underscoring the potential utility of eosinophil monitoring in the management of such allergic reactions. To date, the precise mechanism by which meropenem induces DTH remains poorly understood. The hapten hypothesis described that small molecular sunch as *β*-lactams lack immunogenicity but possess hapten potential (5, 16). Their *β*-lactam ring structures are prone to covalently bind to lysine residues of host serum proteins (such as albumin) via ring-opening reactions, forming stable drug-protein complex that serves as target for capture by antigen-presenting cells (APCs). Previous studies have demonstrated that meropenem can form structurally distinct haptenic complexes with lysine residues on human serum albumin, thereby generating antigenic determinants capable of activating meropenem-specific T-cell responses (17). We hypothesize that this process may contribute to the pathogenesis of meropenem-associated DTH reactions.

**Table 2:** Case reports of meropenem-induced delayed-type hypersensitivity reaction.

Reference	Gender/Age (year)	Infection type	Trigger carbapenem	Adverse reaction latency	Manifestation	Manegement	Outcome of rash
Noguerado-Mellado et al. (9)	Female/61	Sepsis	Meropenem	3–4 days	Generalized scaly erythematous rash (involving oral, vaginal, and rectal mucosa) with subsequent desquamation	Stop meropenem with antihistamine and corticosteroid treatment	Recovered in 25 days
Morgado et al. (10)	Female/38	Abdominal wall cellulitis	Meropenem	11 days	Morbilliform	Replace meropenem with imipenem-cilastin	Recovered
Morgado et al. (10)	Female/61	Postoperative infection	Meropenem	10 days	Maculopapular exanthema	/	Recovered

In our case, meropenem was discontinued and replaced with imipenem-cilastatin as an alternative anti-infective therapy with no observed adverse reactions. Although imipenem-cilastatin and meropenem both belong to the carbapenem class of antibiotics and may potentially cause cross-reactivity adverse effects, clinical practice has demonstrated that they can serve as safe alternatives to each other in certain cases (Supplementary Table S1). Unlike imipenem, meropenem features a methyl group substituent at position 1 and a pyrrolidine-3-thiol substituent at position 2 of its structure. We hypothesize that this structural differences in their side chains could lead to variations in their pharmacological properties. Furthermore, meropenem-induced hypersensitivity reactions may result from immune system interactions with its metabolites rather than the native drug molecule itself. The potentially greater structural divergence between meropenem metabolites and imipenem-cilastatin may significantly reduce cross-reactivity risks. To our knowledge, only one previous case report has documented similar meropenem-induced DTH and represented successful application of imipenem-cilastatin as an alternative therapeutic agent. Our case offers valuable guidance for clinicians managing patients who develop comparable meropenem-induced DTH and still require effective infection control.

## 4 Conclusion

This case report highlights the potential of a DTH following meropenem treatment. Our practical experience provides an effective management strategy for this adverse reaction, suggesting that imipenem-cilastatin can be an effective alternative for infection control in patients allergic to meropenem. Notably, meropenem-induced DTH is uncommon in clinical practice. Further investigation on the precise underlying mechanisms is warranted to optimize prevention and therapeutic interventions for this adverse reaction.

## Data Availability

The original contributions presented in the study are included in the article/Supplementary material, further inquiries can be directed to the corresponding author.
