# Supplementary material for: Case Report: Delayed-type hypersensitivity reaction to meropenem in an elderly patient—successful management with imipenem-cilastatin
Source: Front Med (Lausanne). 2025 Oct 29;12:1671455. doi: 10.3389/fmed.2025.1671455 (PMC12605085; doi:10.3389/fmed.2025.1671455)
Supplement: Supplementary file 1 [file Table_1.docx]

**Supplementary materials**

**
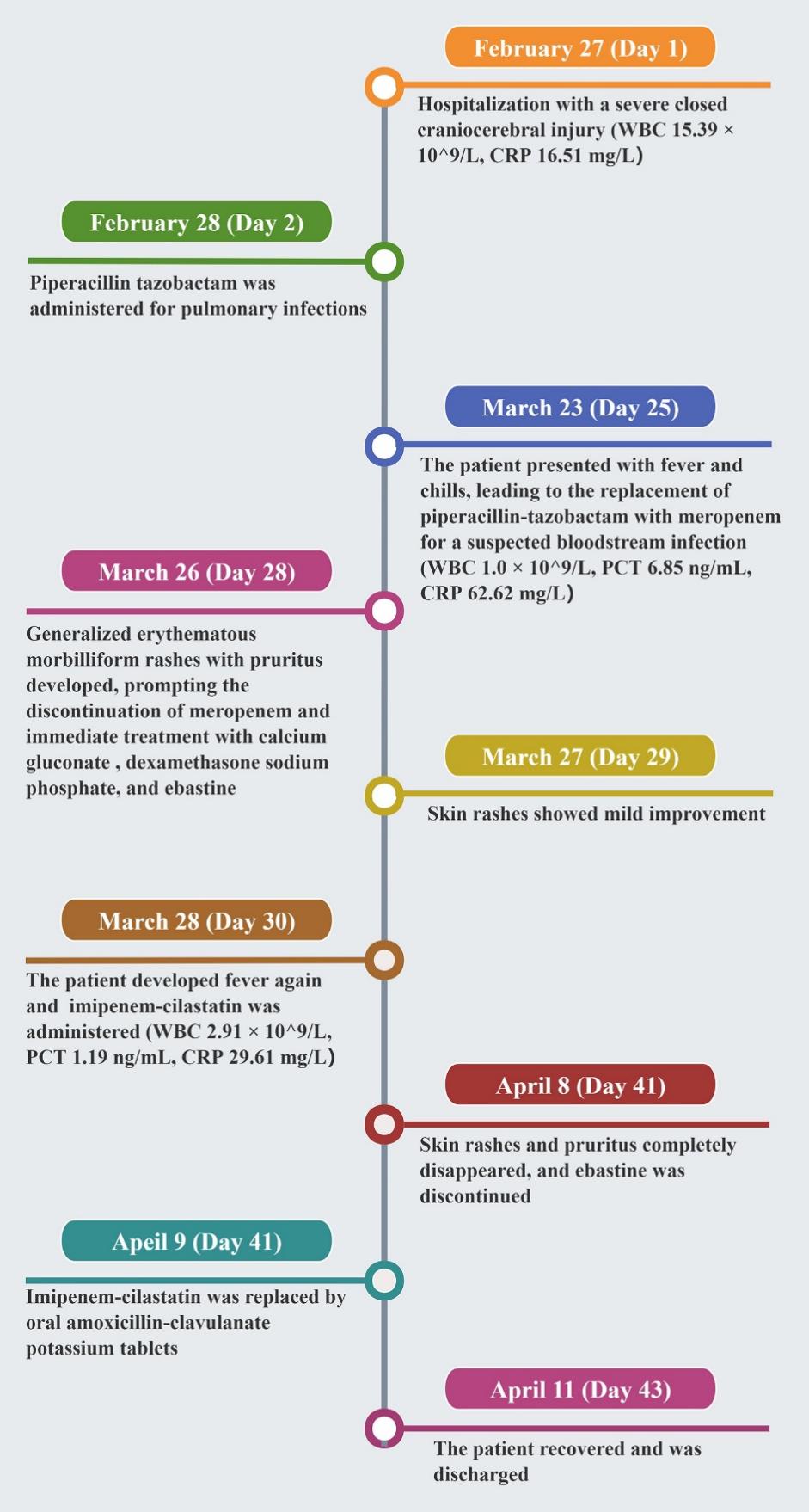
**

**Figure S1. Disease progression and management timeline.**

**Table S1. Case reports of cross-reactivity between meropenem and imipenem-cilastatin**

| **Reference** | **Gender/Age (year)** | **Infection type** | **Trigger carbapenem** | **Adverse reaction latency** | **Manifestation** | **Alternative carbapenem** | **Outcome of rash** | **Cross-reactivity^*^** |
| --- | --- | --- | --- | --- | --- | --- | --- | --- |
| Blanca et al., 2014 (1) | Female/61 | Sepsis | Meropenem | 3-4 days | Generalized scaly erythematous rash (involving oral, vaginal, and rectal mucosa) with subsequent desquamation | / | Recovered | None (meropenem: +, imipenem-cilastatin: –) |
| Morgado et al., 2020 (2) | Female/38 | Abdominal wall cellulitis | Meropenem | 11 days | Morbilliform | Imipenem-cilastin | Recovered | None (meropenem: +, imipenem-cilastatin: –) |
| Morgado et al., 2020 (2) | Female/61 | Postoperative infection | Meropenem | 10 days | Maculopapular exanthema | / | Recovered | None (meropenem: +, imipenem-cilastatin: –) |
| Bauer et al., 2004 (3) | Female/41 | Septic shock | Imipenem-cilastatin | 2 days | Erythematous maculopapular rash with areas of urticaria on pannus, buttocks, and legs | Meropenem | Recovered | None (clinical observation) |
| Karim et al., 2007 (4) | Female/48 | Ventilator-associated pseudomonas aeruginosa pneumonia | Imipenem-cilastatin | 8 days | Extensive erythematous macular morbilliform rash | Meropenem | Recovered | None (meropenem: –, imipenem-cilastatin: +) |
| Randa et al., 2024 (5) | Male/50 | Parietal wall abscess | Imipenem-cilastatin | 3 days | Generalized itching maculopapular exanthema | Ertapenem | Recovered | None (meropenem: –, imipenem-cilastatin: +) |

**^*^**Cross-reactivity results are obtained by skin tests ('+' indicates a positive result, '–' indicates a negative result) or clinical observations.

**References**

1. Noguerado-Mellado B, Pinto Fernández C, Pineda-Pineda R, Martínez Lezcano P, Álvarez-Perea A, De Barrio Fernández M. Cross-reactivity between carbapenems: two case reports. J Allergy Clin Immunol Pract. 2014;2(6):816-7. doi.org/10.1016/j.jaip.2014.06.015.

2. Morgado F, Santiago L, Gonçalo M. Safe use of imipenem after delayed hypersensitivity to meropenem-Value of patch tests. Contact Dermatitis. 2020;82(3):190-1. doi.org/10.1111/cod.13435.

3. Bauer SL, Wall GC, Skoglund KJ, Peters LK. Lack of cross-reactivity to meropenem in a patient with an allergy to imipenem-cilastatin. J Allergy Clin Immunol. 2004;113(1):173-5. doi.org/10.1016/j.jaci.2003.10.055.

4. Lakhal K, Lortat-Jacob B, Neukirch C, Pajot O, Wolff M. Safe use of meropenem in a patient with a possible nonimmediate allergy to imipenem. Pharmacotherapy. 2007;27(9):1334-8. doi.org/10.1592/phco.27.9.1334.

5. Laajimi R, Mansour K, Chadli Z, Ben Fredj N, Ben Romdhane H, Chaabane A, et al. Tolerability of Meropenem and Ertapenem in a Patient with Documented Delayed Hypersensitivity Reaction to Imipenem-Cilastatin: A Literature Review. Dermatitis. 2025;36(1):e89-e92. doi.org/10.1089/derm.2024.0059.
